# HAC stability in murine cells is influenced by nuclear localization and chromatin organization

**DOI:** 10.1186/1471-2121-10-18

**Published:** 2009-03-06

**Authors:** Daniela Moralli, David YL Chan, Andrew Jefferson, Emanuela V Volpi, Zoia L Monaco

**Affiliations:** 1 Wellcome Trust Centre for Human Genetics, University of Oxford, Roosevelt Drive, Oxford, OX3 7BN, UK

## Abstract

**Background:**

Human artificial chromosomes (HAC) are small functional extrachromosomal elements, which segregate correctly during each cell division. In human cells, they are mitotically stable, however when the HAC are transferred to murine cells they show an increased and variable rate of loss. In some cell lines the HAC are lost over a short period of time, while in others the HAC become stable without acquiring murine DNA.

**Results:**

In this study, we linked the loss rate to the position of the HAC in the murine cell nucleus with respect to the chromocenters. HAC that associated preferentially with the chromocenter displayed a lower loss rate compared to the HAC that are less frequently associated. The chromocenter acts as a hub for the deposition of heterochromatic markers, controlling centromeric and pericentromeric DNA replication timing and chromosome segregation. The HAC which localized more frequently outside the chromocenters bound variable amounts of histone H3 tri-methylated at lysine 9, and the high level of intraclonal variability was associated with an increase in HAC segregation errors and delayed DNA replication timing.

**Conclusion:**

This is a novel result indicating that HAC segregation is closely linked to the position in the murine nucleus and gives important insight for HAC gene expression studies in murine cells and establishing murine models of human genetic disease.

## Background

Human artificial chromosomes (HAC) are small autonomous extrachromosomal elements, which replicate and segregate correctly as stable chromosomes [1]. De novo HAC are generally produced following introduction of centromere specific DNA into target cells. The HAC have been used successfully to understand the chromatin organization and epigenetic status of human centromeres [1-3], and the structure of the kinetochore [4].

In human cells, the HAC are generally stable, and are maintained by the cells in absence of selection over prolonged time in culture [5-9]. When compared to the other human chromosomes however, they display a slight increase in segregation errors, possibly due to the de novo HAC structure [5].

HAC also represent ideal vectors for gene expression studies in mammalian cells [1]. They can be formed directly in murine cells, by introduction of human alpha satellite DNA and are mitotically stable [2]. However, the majority of studies characterizing HAC segregation and gene expression pattern have involved the transfer of HAC to rodent cells via microcell mediated chromosome transfer (MMCT) [6-9]. In these experiments, both de novo HAC and artificially size-reduced minichromosomes showed variability in the stability of different HAC in murine cells, from stable to highly unstable [6-10]. Generally, the HAC differential stability has been attributed to differences in structure (e.g., circular versus linear HAC [10]) or composition [7,9]. Our studies focus on the relationship between organization of the cell nucleus and HAC stability. In murine cultured cells, the nucleus is characterized by a compartmentalized distribution of chromosome regions. In particular, the major satellite rich pericentromeric regions from several chromosomes pool together to form the chromocenter [11], a DAPI positive, highly condensed nuclear area [11]. The centromeres, formed on a repetitive sequence called minor satellite [12], localize at the periphery of the chromocenters [11]. The nuclear organization is important for the correct replication timing and deposition of histone-specific modifications, such as the trimethylation at lysine 9 of histone H3 (H3triK9) which is associated with heterochromatin. The H3triK9 has been linked to several fundamental processes for chromosome stability, such as DNA replication timing [13], the deposition of the centromere specific histone CENP A [14], the maintenance of chromatid association until anaphase [11] and chromosome condensation [15].

In this report we undertook a comparative study to analyze differences between the HAC in murine cells with respect to human cells, and characterized the differential stability displayed by the HAC in human and murine backgrounds. We investigated two different HAC, generated in HT1080 cells following lipofection [16,17], and their corresponding derivatives in murine cells following transfer by microcell mediated chromosome transfer (MMCT) [8]. The analysis included characterizing the HAC chromatin organization, the binding of centromeric protein A (CENP A), and monitoring HAC stability. There was no significant difference in the CENP A binding capacity of the HAC when compared to the endogenous host chromosomes in human and mouse cells. However, the HAC loss rate in the absence of selection was different between clones, and was higher in the murine cells with respect to the parent HAC in human cells. Possible causes for the differential stability were investigated. A statistically significant correlation was detected between the position of the HAC in the murine nucleus to the loss rate, as the HAC were more stable when they were localized partially or completely within a chromocenter. The variation in the amount of H3triK9 bound by the HAC was similarly correlated to nuclear position and loss rate. This raised the interesting hypothesis that in murine cells the difference in HAC stability observed in this study and in the literature [6-10] may be affected by topological positioning in the nucleus, as well as intrinsic differences in HAC structure.

## Results

### HAC structural analysis

**Figure 1:**
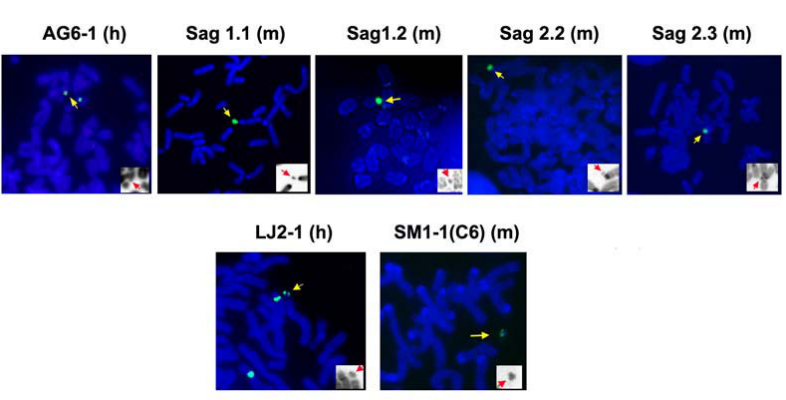
**FISH on metaphase spreads from HAC containing cells lines in human and murine cells**. Metaphase spreads from all HAC clones were hybridized to 17α DNA (green signal). The HAC in human cells (h) are grouped with the derivative HAC in murine cells (m). Chromosomes are counterstained by DAPI (blue). The yellow arrows denote the HAC. The inset shows DNA staining only, with HAC identified by the red arrow.

**Figure 2:**
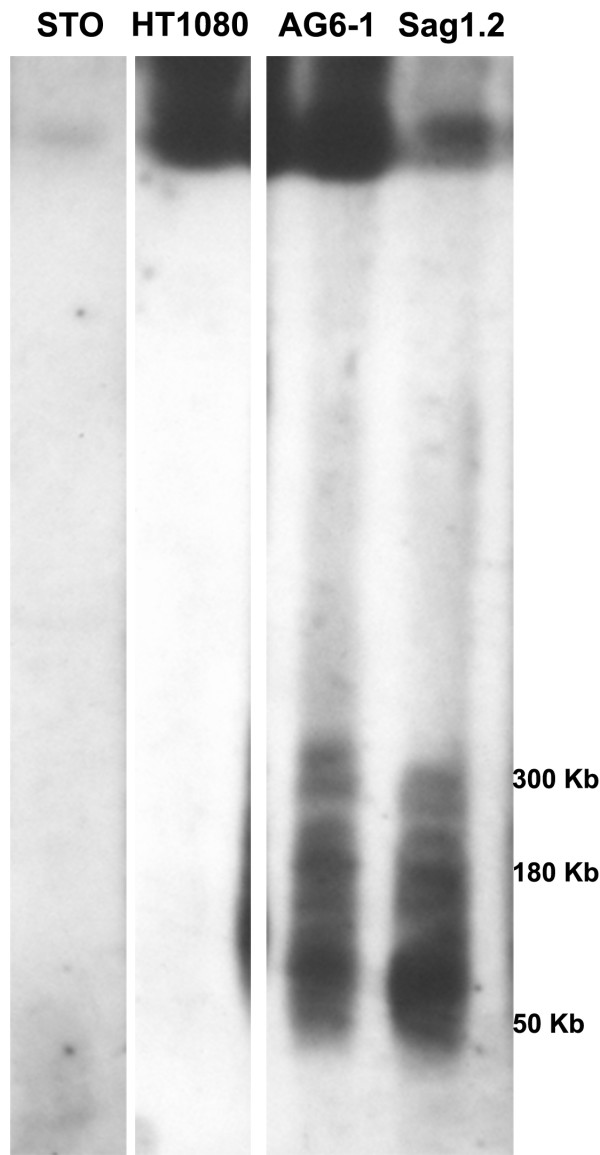
**PFGE analysis of HAC clones**. Genomic DNA from the HAC containing cell lines AG6-1 (HT1080), and its murine derivative HAC Sag1.2 (STO) and the parental HT1080 and STO cells was digested with Kpn I, fractionated by PFGE and hybridized to a 17α probe. The apparent difference in fragment sizes is due to gel distortion.

The HAC used included the LJ2-1 containing 17α DNA and generated in human HT1080 cells [17], and the murine derivative HAC SM1-1 (C6) generated after MMCT transfer of LJ2-1 in STO cells [8]; HAC AG6-1, containing 17α DNA and the HPRT genomic locus, generated in HT1080 [16], and the murine derivative HAC Sag 1.1, Sag1.2, Sag2.2 and Sag 2.3, generated in this study after MMCT transfer of AG6-1 HAC in STO cells. The MMCT between AG6-1 and STO led to the isolation of 9 independent clones, corresponding to an efficiency of 2.25 × 10^-5^. Four clones containing the HAC were chosen for further analysis as they did not contain any other human chromosome or chromosome fragment as seen by FISH with total human DNA (data not shown). Figure 1 shows FISH on metaphase spreads of HAC in each cell line used in this study. All clones contained on average one HAC per cell (Figure 1). Neither HAC SM1-1 (C6) [8], nor HAC Sag1.1, Sag1.2, Sag2.2, Sag2.3 contained any murine DNA, as demonstrated by FISH with mouse genomic DNA and mouse satellite DNA (data not shown). The HAC LJ2-1 and SM1-1 (C6) were characterized previously by PFG analysis and found to have different structures, indicating that the HAC underwent a rearrangement following MMCT transfer to STO cells [8]. A similar analysis was undertaken to study the structure of human HAC AG6-1 and its murine derivative HAC Sag1.2. Following digestion with Kpn I and hybridization with a 17α probe, no major differences were detected between the HAC AG6-1 and Sag1.2 (Figure 2), Sag1.1, Sag2.2 and Sag 2.3 (data not shown) suggesting that if a rearrangement had occurred during MMCT transfer of AG6-1 HAC to murine cells, it was less pronounced than that observed in HAC SM1-1 (C6).

### Centromere protein A and chromatin histone analysis

**Figure 3:**
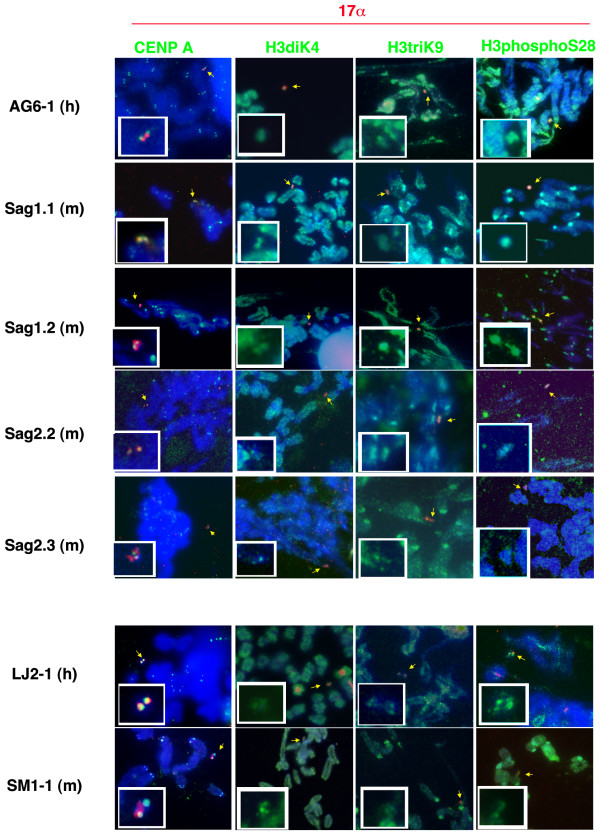
**Immuno-FISH on metaphase spreads from HAC containing cells lines in human and murine cells**. The human HAC are grouped with their murine derivatives. HAC are stained with antibodies for CENP A, H3diK4, H3triK9 and H3phosphoS28 (green signal) and hybridized by FISH with the 17α probe (red signal). The chromosomes are counterstained in DAPI (blue). The small white inset shows a 3× enlargement of the HAC identified by the yellow arrows. To appreciate the amount of H3diK4, H3triK9 and H3phosphoS28 bound by the HAC, the FISH signal is not displayed in the corresponding white inset.

Chromosome metaphase spreads containing the HAC were stained with specific antibodies to the essential centromere protein A (CENP A), as well as to histone H3 dimethylated in Lysine 4 (H3diK4) and H3triK9. The histone modifications mark respectively the DNA as euchromatic or heterochromatic [3,18], and it has been shown that while the HAC generally bound H3diK4 (euchromatin), the H3triK9 (heterochromatin) is sometimes absent [19]. We also investigated the presence of histone H3 phosphorylated in Serine 10 or 28 (H3phospoSer10 and H3phosphoSer28), as these modifications produced by Aurora B kinase activity are probably required for chromosome condensation before mitosis and regulation of DNA transcription [20]. The HAC were identified by simultaneous hybridization with 17α DNA probes (Figure 3). All the HAC bound CENP A protein, and were labeled by H3diK4, H3phospoSer10 (data not shown) and H3phosphoSer28 at a level similar to the other chromosomes (Figure 3). The H3triK9 is typically associated with constitutive heterochromatin and transcriptionally silenced DNA, and in mouse chromosomes is most prevalent in the pericentromeric area. All HAC analyzed in this study bound the anti-H3triK9 antibody (Figure 3) as well as H3diK4, indicating that they comprised of both heterochromatin and euchromatin respectively.

### Mitotic stability

The stability of all HAC was monitored for at least 150 days in absence of selection by FISH analysis with HAC specific probes on metaphase spreads. The HAC segregation at 0 and 150 days, and the daily loss rate are shown in Table 1. All the parental HAC in human cells were stable (daily loss rate of 0.04–0.07%), whereas all the derivative HAC in murine cells exhibited an increased level of instability. Among the murine HAC the most stable was Sag1.1 with 80% of HAC present at day 0 and 20% of HAC present after 150 days. Conversely, the HAC showing the highest instability was SM1-1 (C6), which at day 0 was 100%, but was completely lost after 30 days of selection [8]. There was no correlation between the loss rate of the parental HAC and that of the derivative HAC in murine cells. The HAC displaying the highest loss rate (SM1-1 (C6)) was derived by LJ2-1, whose loss rate in human HT1080 cells was the lowest (0.04%). Murine HAC were characterized by different degrees of stability among the Sag clones derived from the same human parental line. We thus investigated possible causes for the observed behavior, focusing in particular on nuclear architecture, that appear to be strictly regulated in murine cells [11].

**Table 1:** HAC daily loss rate

	**HAC (%) 0 days off selection**	**HAC (%)** **150 days off selection**	**Loss rate** **(%)**
**17α-HPRT HAC**			
			
AG6-1 (h)	96	86	0.07
Sag1.1 (m)	80	20	0.92
Sag 1.2 (m)	85	6.6	1.68
Sag 2.2 (m)	88	6	1.77
Sag 2.3 (m)	88.6	10	1.44

**17α-HAC**			
			
LJ2-1 (h)	92	86	0.04
SM1-1(C6) (m)	100	Nd	*5.20

The percentage of HAC containing metaphases in human (h) and murine derivatives (m), in absence of selection at 0 and 150 days is shown. The last column on the right indicates the percentage loss rate (%) after 150 days in the absence of selection, calculated by the formula *N*_*n *_= *N*_0 _*× (1-R)*^*n*^, where *N*_0 _is the number of metaphase chromosome spreads showing HAC in the cells cultured under selection, *N*_*n *_is the number of HAC-containing metaphase chromosome spreads after n days of culture in the absence of selection, and *R *is the daily rate of loss. Each table cell shows the human parentals (at the top) and their mouse derivatives. *nd: *none detected

* Loss rate after 30 days [8].

### HAC localization in the nucleus

**Figure 4:**
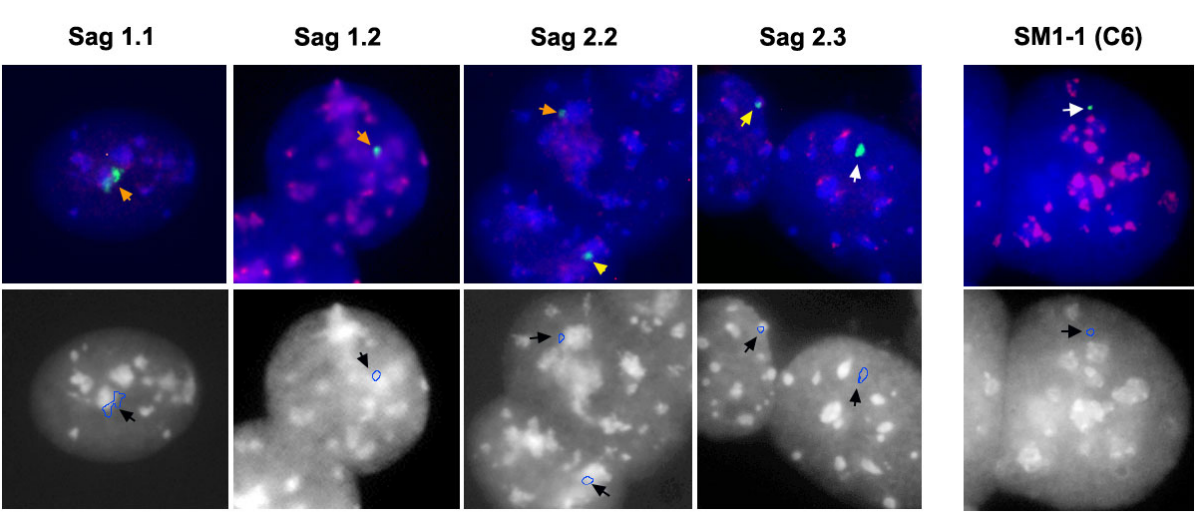
**HAC colocalization with the chromocenters**. Top panels: cells from each of the murine HAC clones were hybridized with 17α (green signal) and minor satellite probes (red signal). In this experiment, the chromocenters are identified by the typical high staining with DAPI (blue). In some cells the HAC do not colocalize with the chromocenters (white arrow), while in other cells there may be partial (orange arrow) or complete colocalization (yellow arrow). The bottom panels display the same cells, stained with DAPI to show the chromocenters distribution, and the outline of the HAC in blue (black arrow).

To determine if HAC position in the nucleus had an influence on HAC stability, the percentage of HAC localizing with the murine chromocenters was correlated to HAC loss rate in the absence of selection. FISH was carried out with a HAC specific, a major satellite and a minor satellite probes. A total of 150 interphase cells from HAC SM1-1 (C6), Sag1.2, Sag1.1, Sag2.1 and Sag 2.2 were analyzed for each clone, and the position of the HAC relative to the DAPI positive, major satellite rich chromocenters, was scored. The results are shown in Table 2, where all clones are characterized by the percentage of association between the HAC and the chromocenters. The HAC Sag1.1 showed the highest level of association (30.5%) with the chromocenters, and SM1-1 (C6) the lowest (0%). A similar analysis was carried out to determine if HAC colocalize with mouse centromeric regions, as identified by FISH with minor satellite sequences (Figure 4, Additional file 1). HAC colocalizing with minor satellite sequences were scored as being outside the chromocenter, since murine centromeres are localized at the periphery of these structures [11]. The data are presented in Table 2. HAC SM1-1 (C6) displayed the highest level of colocalization (24.97%) with the centromeres, while HAC Sag2.2 was the lowest (5.04%). To determine if incorrect localization in the nucleus can lead to an increase in HAC instability, the percentage of HAC localizing with the chromocenters or the centromeres was correlated to HAC loss rate over 150 days in absence of selection. A statistically significant inverse correlation (p = 0.0114, Pearson Product-Moment Correlation r = -0.9282) was observed between HAC position with respect to the major satellite (chromocenters) and loss rate. No correlation was found between colocalization with minor satellite and loss rate (p = 0.0878) suggesting that HAC not associated with a chromocenter have a higher loss rate, as in SM1-1 (C6), while colocalization with mouse centromeric sequences is possibly less relevant.

**Table 2:** HAC nuclear localization

**HAC clone**	**Colocalization with Chromocenters (%)**	**Colocalization with Centromeres (%)**	**Daily loss rate** **(%)**
Sag1.1	30.50	6.08	0.92
Sag 1.2	14.60	21.25	1.68
Sag2.2	21.76	5.04	1.77
Sag 2.3	20.34	13.70	1.44

SM1-1 (C6)	0	24.97	5.22

The frequency of HAC colocalizing with a chromocenter or mouse centromeric region in murine clones.

### The H3triK9 analysis

Major satellite chromatin, which mainly localizes in the chromocenter, is heavily modified by H3triK9. To determine the importance of the chromatin modification for HAC segregation in murine cells, metaphase spreads from all the clones were stained with anti H3triK9 antibody (Figure 3), and the average fluorescence levels of each HAC quantified in 25 metaphases using ImageJ software. The values were normalized for difference in HAC size and in antibody binding efficiency between metaphases with respect to mouse chromosome 19. The HAC H3triK9 values are expressed using the formula: (HAC H3trik9/HAC DAPI)/(mouse chromosome 19 H3triK9/mouse chromosome 19 DAPI). The data are reported in Table 3. On average, all HAC bound H3triK9 antibody at a level similar (albeit slightly higher) to the murine host chromosomes (ratio values between 1.157–1.3922). There is a narrow correlation between average amount of H3triK9 on HAC per clone, and loss rate (p = 0.0534), suggesting that the larger the ratio of H3triK9 HAC/H3triK9 mouse chromosomes, the less stable is the HAC. However, when the data are analyzed at the level of a single metaphase spread, some clones display a higher intraclonal variability in the amount of H3triK9 bound by HAC in different cells. For example, the H3triK9 HAC/H3triK9 mouse chromosomes ratio measured in different cells of clone Sag1.1 is fairly consistent, varying from a minimum of 1.182 to a maximum of 1.45 (data not shown. Table 4 lists the average ratios and the standard deviation value). In clone Sag2.3 the intraclonal variability is higher, with H3triK9 HAC/H3triK9 mouse chromosomes ratio values ranging between 0.722 and 1.655. Clone SM1-1 (C6) shows the highest intraclonal variability, with values varying from 0.535 to 2.5. The intraclonal differences in H3triK9 HAC/H3triK9 mouse chromosomes ratio values are measured by the differences in standard deviation between clones (Table 4). The variability in amount of H3triK9 present on HAC in different metaphases directly correlates with the loss rate (p = 0.0066; r = 0.9502), suggesting that the less homogeneous is H3triK9 deposition on HAC in different cells of the same clone, the higher the instability of the HAC. Finally, the variability of H3triK9 binding, as measured by the standard deviation, significantly inversely correlates with the HAC position in the nucleus with respect to the chromocenters, (p = 0.0066; r = -0.951).

**Table 3:** Histone H3triK9 quantization

**Murine HAC**	**H3triK9** **(Mean)**	**H3triK9** **(Standard deviation)**
Sag1.1	1.2418	0.0812
Sag 1.2	1.2237	0.2110
Sag 2.2	1.3166	0.2107
Sag 2.3	1.1570	0.2628

SM1-1 (C6)	1.3922	0.5076

Relative amount of histone H3triK9 bound by the HAC in murine clones, measured as the ratio [HAC H3trik9/HAC DAPI]/[mouse chromosome 19 HtriK9/mouse chromosome 19 DAPI].

**Table 4:** CBMN analysis of HAC segregation errors in murine cells.

**HAC**	**HAC non-disjunction events (%)**	**HAC loss events (%)**
Sag1.1	6.77	0
Sag1.2	11.40	0
Sag2.2	18.00	0
Sag2.3	11.86	1.7

SM1-1 (C6)	23.40	0

### The frequency of non-disjunction events in HAC clones

The H3triK9 modification has been associated with several important cellular processes, including maintenance of chromatid pairing during mitosis. In murine cells, the sister chromatids maintain an attachment corresponding to the H3triK9 rich major satellite pericentromeric region, after the chromosome arms and minor satellite centromeric region have already been separated [11]. Thus, it is likely that perturbation in the levels of H3triK9 could lead to an increase in segregation errors, most likely due to problems with disjunction events. To verify how frequently HAC in the different murine clones are involved in segregation errors, we carried out a cytokinesysis block micronucleus assay (CBMN) that measured the incidence of chromosome loss and non-disjunction. All HAC containing cell lines were treated with cytochalasin B to stop cytokinesis after cell duplication, and then the cells were analyzed by FISH with a HAC specific probe. At least 60 binucleated cells were scored for non-disjunction events, i.e. showing an unbalanced number of FISH signals in the two daughter cells. The presence of HAC containing micronuclei was scored as a chromosome loss event. The results are presented in Table 4. Chromosome loss events were detected at very low level (1.7%) only in clone Sag2.3. All the clones displayed different levels of non-disjunction events, from 6.77% (Sag1.1) to 23.4% (SM1-1 (C6)). There is significant direct correlation between loss rate and non-disjunction events (p = 0.0261; r = 0.875), and H3trik9 variability (as measured by the standard deviation in table 3) and non-disjunction events (p = 0.0255; r = 0.877). Overall our data suggested that segregation errors, and more specifically non-disjunction events, played an important role in HAC loss in murine cells.

### HAC replication timing

**Figure 5:**
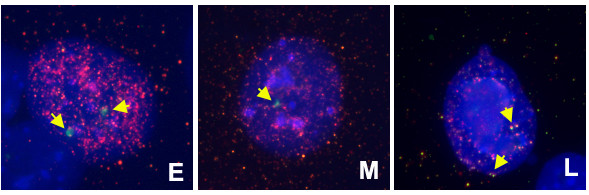
**Analysis of HAC replication timing**. To identify the S phase stage, the cells from clone Sag1.2 were stained with an anti-BrdU antibody (red signals). HAC were identified by FISH with a 17α probe (green signal). DNA was counterstained with DAPI (blue). Examples of early (E), mid (M) and late (L) replicating HAC are shown.

To investigate whether there were differences in replication timing of HAC in different clones, cells were synchronized with aphidicolin and then released in the presence of the base analogue BrdU, to mark newly replicating DNA. Cells were harvested at 3 hours (early S), 6 hours (mid S) and 9 hours post-release (late S), and hybridized to a HAC specific probe. In murine cells, each stage of the S-phase corresponds to a distinct BrdU labeling pattern [11] so that each stage was easily identified (Figure 5). In human cells, tri-color FISH experiments were carried out using a probe for a euchromatic, early replicating locus (*HPRT*) one for a heterochromatic, mid to late replicating sequence (17 alpha satellite) and the HAC specific probe (vector DNA, pBeloBAC11). Doublet signals were considered to correspond to the two replicated chromatids, while singlet signals were scored as unreplicated DNA. The results are summarized in Table 5. The human HAC replicated mostly in mid-S phase (AG6-1, 51.6%, LJ2-1 77%), along with the chromosome 17 centromeric region. In murine cells, the majority of HAC replicated between early and mid S. The HAC Sag 2.3, however, replicated mainly during mid S. On the other hand, HAC SM1-1 (C6), which displayed a high loss rate in murine cells, replicated more frequently in late S-phase (60%). There is a direct correlation between late S phase replication and higher loss rate of HAC in murine clones (p = 0.0343; r = 0.849).

**Table 5:** HAC replication timing

**HAC**	**Early S (%)**	**Mid S (%)**	**Late S (%)**
AG6-1 (h)	15.0	51.6	33.3
Sag1.1 (m)	28.8	40.0	31.1
Sag 1.2 (m)	25.0	29.2	45.8
Sag2.2 (m)	29.6	48.1	23.0
Sag2.3 (m)	7.5	32.0	41.0

LJ2-1 (h)	15.4	77.0	7.6
SM1-1 (C6) (m)	4.3	27.2	68.5

The Table shows the percentage of HAC (human (h) and murine derivative (m)) that are replicating at each stage (early, mid and late) of cell cycle S phase in each cell line.

## Discussion

Since the 1960s, when inter-specific somatic cell hybrids were first described [21], the progressive loss of human chromosomes in murine cells has represented a puzzling problem. In some of the hybrids, most chromosomes were lost and only a few displayed a higher stability without acquiring murine DNA [22]. More recently, studies on artificially size reduced minichromosomes and HAC in rodent cells, have shown large differences in stability between clonal lines, even when they are derived from the same parent [6-8]. In some cases [7], the minichromosomes lacked essential centromere and kinetochore components, thus explaining the high loss rate observed in murine cells in absence of selection. In most other cases, however, the essential proteins CENP C and CENP A were present at apparently normal levels, but the HAC or minichromosome still displayed high levels of instability [6,8].

In this study, we analyzed the chromatin organization and segregation behavior of two HAC in human and murine cells, with the aim of identifying factors important for correct chromosome function. The human HAC AG6-1, containing 17α DNA and the *HPRT *genomic locus [16], and LJ2-1, containing 17α DNA only [17] were transferred to murine cells by MMCT. HAC Sag 1.1, Sag 1.2, Sag 2.2, and Sag 2.3 (derived from HAC AG6-1) and SM1-1 (C6) (derived from HAC LJ2-1) respectively were generated. To rule out that differences between HAC in murine cells could be the effect of biochemical variations between cell lines, STO cells were used for both experiments. They are immortalized embryonal fibroblast cells derived from the SIM mouse, and are often used as feeder cells for murine and human ES cell culture [23].

To determine the possible causes for the differential stability displayed by the HAC in the murine cells, we first investigated if the HAC bound CENP A, a protein that plays a key role in centromere formation and chromosome segregation. All the HAC bound CENP A in an amount similar to each other and to the human or murine endogenous chromosomes. We then characterized the histone modification associated with either euchromatin (H3diK4) or heterochromatin (H3triK9). All HAC, in both human and murine cells, were euchromatic in composition, but also contained a heterochromatic domain. Similarly, the histone H3 modifications associated with chromosome condensation prior to mitosis (H3phosphoSer10/Ser28) were also present on all HAC.

We investigated the nuclear positioning of the HAC in murine cells as it is important for key processes such as DNA replication, and chromosome segregation. A statistical analysis of the HAC position in the nucleus of STO cells was undertaken on SM1-1 (C6), Sag1.1, Sag1.2, Sag 2.2, and Sag 2.3. In all the murine clones analyzed, the HAC displayed a differential level of stability, even when deriving from the same parental HAC (Sag clones). The HAC Sag 1.1 was the most stable (loss rate 0.92%) and SM1-1 (C6) was the least stable (loss rate 5.2%). The position of the HAC in the nucleus, and more specifically the association with the chromocenters correlated with HAC stability. The HAC that associated more frequently with the chromocenters i.e. Sag 1.1, Sag 2.2 and Sag 2.3, have a lower loss rate compared to SM1-1 (C6), which was not localized with the chromocenter. The data show that the positioning of HAC in the nucleus of murine cells was important for correct chromosome segregation. Also, the HAC that colocalize less frequently with chromocenters displayed intraclonal variable levels of H3triK9 in different cells. The H3triK9 variability in turn, correlated significantly with the loss rate. The H3trik9 modification is fundamental for maintenance of chromatid pairing at the centromere [7,11], and it is linked to determination of the replication timing. The frequency of segregation errors (non-disjunction events) in the murine HAC clones correlated significantly with the H3triK9 variability. The HAC in murine cells tended to replicate late in the S phase, which correlated with increased HAC loss rate. It is possible that this behavior is linked to the incorrect epigenetic marking of the HAC, however there is no direct evidence to support this. It may be that the human HAC replication origins are not fully functional in murine cells, or are recognized less efficiently. The murine origins of replication would compete for the replication factors machinery, that hence would become accessible to the HAC sequences only when most of the host DNA has been replicated.

Although a significant statistical correlation does not indicate that there is a causality link between two variables, based on the data we obtained, it seems likely that when a high percentage of HAC do not associate with the chromocenter, they are removed from the nuclear environment that allow the deposition of the correct heterochromatic marker. The resulting variability in the H3triK9 deposition is responsible for the high loss rate displayed by HAC in murine cells, due to segregation errors and/or incorrect replication timing. On the other hand, it is possible that the more stable HAC are the ones that in the initial replications, after the MMCT, assembled the correct levels of heterochromatin, and because of that they then localized in the heterochromatin rich chromocenter. However, recent publications, that show the existence of a pericentric heterochromatin duplication body [24,25], lend support to the theory that it is necessary for the HAC to be localized within the chromocenter to guarantee that they are replicated at the correct time, and are modified with the appropriate heterochromatin markers.

But what is the mechanism that leads some of the HAC to localize within a chromocenter while others do not? The chromocenter structure is re-formed, after each cell division, in the early G1 phase [24]. It is possible then that the different murine HAC clones were generated by MMCT in cells in different phases of the cell cycle, and synchronization of the receiving murine cells may be important in determining if this is the case. Alternatively, other unknown factors may be responsible for the difference in nuclear positioning of the HAC. The HAC SM1-1 (C6) characterized by the highest loss rate, is composed mostly of 17α DNA, while the other murine HAC, derived from AG6-1 also contains large non-satellite sequences corresponding to the human HPRT genomic locus. The different composition may play a role in determining the localization of the HAC in the nucleus. For larger human chromosomes, there seems to be a preferential positioning in the murine nucleus related to gene richness [26], or to mimic the position of the mouse syntenic chromosomes [27]. It is possible that the HAC analyzed in this study are too small, or have no syntenic correspondent in the mouse genome and so are randomly allocated in the nuclear structure. Also, we cannot rule out that rearrangements in the HAC chromosome structure during the MMCT procedure (such as in HAC SM1-1 (C6) following transfer from LJ2-1) may interfere with epigenetic markings conferring its heterochromatic characteristics to the HAC pericentromeric region, and as a result the HAC are not correctly positioned in the mouse nucleus. All of the HAC characterized in this study are most likely circular [16]. Thus, topological constraints may have an effect on their stability.

Finally, it is possible that protein components of the murine kinetochore are not fully capable of recognizing the human satellite sequences, and thus formed a partially functional centromere which was responsible for the HAC loss. However, the data obtained by the direct generation of HAC in murine cells [2], suggested that, at least in this case, the HAC were able to form a centromere/kinetochore structure and segregate correctly. On the other hand, the stability of these HAC constructs could be due to unknown, specific characteristic of the murine receiving parental. In this light, other reports [9,10] that show differential stability of HAC transferred to murine ES cells could be explained by genetic differences in the mouse strain from which the ES derive.

## Conclusion

In summary, the results indicated that the HAC in human and murine cells were generally organized and behaved like the endogenous host chromosomes, and there was no difference in binding of CENP A between the HAC generated in human or murine cells. However, the HAC were less stable and displayed variable loss rates in murine cells. In a novel result, we linked the variation in stability to incorrect positioning in the nucleus, thus demonstrating that nuclear architecture has a direct effect on chromosome segregation. This effect may also be significant in unidirectional chromosome loss, which has been observed in murine-human interspecific cell hybrids [21]. The study will have important implications for establishing a HAC gene expression system in murine cells and animal models for human genetic disease.

## Methods

### HAC containing cell Lines

Human HAC AG6-1 was generated in the human cell line HT1080 deficient for *HPRT *following lipofection of a 404 Kb BAC containing 220 Kb of chromosome 17 alpha satellite DNA (17α), the G418 and puromycin resistance genes and the human *HPRT *genomic locus [16]; human HAC LJ2-1 was generated in HT1080 cells following lipofection of a BAC containing 240 Kb of 17α DNA and the G418 and puromycin resistance genes [17]. The HAC SM1-1 (C6) was generated in the murine cell line STO [23] (mouse strain SIM) following transfer of the human HAC LJ2-1 via MMCT [8]. The HAC Sag 1.1, Sag1.2, Sag2.2 and Sag 2.3, were generated from the MMCT transfer of HAC AG6-1 in STO cells. All cell lines were cultured in D-MEM medium supplemented with penicillin/streptomycin, 10% FCS and maintained under selection as detailed [8,16,17].

### MMCT

In this study, the human HAC AG6-1 was transferred into murine STO cells by MMCT. Briefly, 10^7 ^donor cells were treated with Colcemid 0.5 μg/ml for 24 hours, harvested, and resuspended in a 1:1 solution of Percoll (Sigma)/DMEM containing Cytochalasin B 10 μg/ml. After a 75 minutes spin at 19.000 rpm, at 25°C, two bands corresponding to the smaller microcells were collected, analyzed at the microscope to rule out the presence of whole cells, counted, washed once in DMEM and mixed to the receiving cells in a 3:1 ratio. The cells were then fused using PEG 50% in PBS (Sigma), and plated. Selection was applied the following day: Puromycin 1 μg/ml, G418 200 μg/ml. After 15–20 days, clones were isolated and expanded.

### Cytological analysis

The FISH and immuno-FISH on metaphase spreads and CBMN analysis were carried out as previously described [16,28]. The antibodies used were as follows: anti-human CENP-A (Abcam); anti-mouse CENP-A (a kind gift from Andy Choo and Paul Kalitsis [29]; anti-histone H3 dimethil lysine 4 (Upstate); anti-histone H3 trimethyl lysine 9 (Abcam); anti-histone H3 phospho serine 10 (Abcam); anti-histone H3 phospho serine 28 (Abcam). All cytological preparations were analyzed using an Olympus BX60 microscope for epifluorescence equipped with a Sensys CCD camera (Photometrics, USA). Images were collected using either MacProbe 4.3 or Genus Cytovision software. Some images were pseudo-colored using Adobe Photoshop 7.0. Fluorescence intensities for H3triK9 staining were measured using the software ImageJ. This was calculated for each metaphase spread by obtaining a value for the ratio between the intensity of HAC H3triK9 signal divided by the intensity of HAC DAPI staining, to normalize for differences in size between images. The same ratio was calculated for the smallest murine chromosome (chromosome 19), and the two ratios were divided to normalize for difference in H3triK9 staining intensities between different metaphase spreads.

### Replication timing analysis

Exponentially growing cells were seeded at 60% confluence in a T25 flask, and kept in DMEM, 0.5% FBS for 24 hours. The medium was then replaced with complete medium (DMEM, 10% FBS), containing 3 μg/ml Aphidicolin (Sigma), and cells were incubated for a further 24 hours. The replication block was released by washing three times in complete medium. The 5-Bromo-deoxy-Uridine (BrdU) (Sigma) was added at a final concentration of 40 μM, at 3, 6 and 9 hours post-release, and left for 15 minutes before harvesting the cells by trypsinization. The cells were briefly swollen in 75 mM KCl, fixed twice in fixative (Methanol:Acetic Acid 3:1), and dropped onto a slide. Following a standard FISH protocol [28] with HAC specific probes (pBeloBAC11 or 17α DNA), BrdU was detected using anti-BrdU antibody (Abcam). Slides were analyzed as described above.

### PFGE

High molecular weight genomic DNA was prepared in agarose plugs, digested and analyzed by PFGE as described [16,17], and hybridized ON at 42°C with a 17α DNA (BAC pJM2256 [17]) probe. Stringent washes were carried out in 0.5 × SSC, 0.1% SDS at 65°C.

## Abbreviations

BAC: Bacterial artificial chromosome; BrdU: 5-Bromo-2-deoxy-uridine; CBMN: Cytokinesis block micronucleus assay; CENP A: Centromere protein A; FISH: Fluorescent in situ hybridization; H3diK4: Histone H3 dimethylated at lysine 4; H3PhosphoSer10: Histone H3 phosphorylated at serine 10; H3PhosphoSer10: Histone H3 phosphorylated at serine 28; H3triK9: Histone H3 trimethylated at lysine 9; HAC: Human artificial chromosome; MMCT: Microcell mediated chromosome transfer; PFGE: Pulse field gel electrophoresis.

## Authors' contributions

All authors read and approved the final manuscript. DM designed experiments, conducted experiments and analyzed data. DYLC carried out experiments and analyzed data. AJ carried out experiments and analyzed data. EVV analyzed data. ZLM designed experiments and analyzed data.

## Supplementary Material

Additional file 1 **HAC colocalization with the chromocenters**. To shows that SM1-1 (C6) do not colocalize with chromocenters (white arrows), but can colocalize with mouse minor satellite (yellow arrows), several cells from this clone are shown, following hybridization with 17α (red signals) and minor satellite probes (green signals). The bottom panels display the same cells, stained with DAPI to show the chromocenters distribution, and the outline of the HAC in blue. Click here for file
