# Supplementary figures and images for: HAC stability in murine cells is influenced by nuclear localization and chromatin organization
Source: BMC Cell Biol. 2009 Mar 6;10:18. doi: 10.1186/1471-2121-10-18 (PMC2674426; doi:10.1186/1471-2121-10-18)

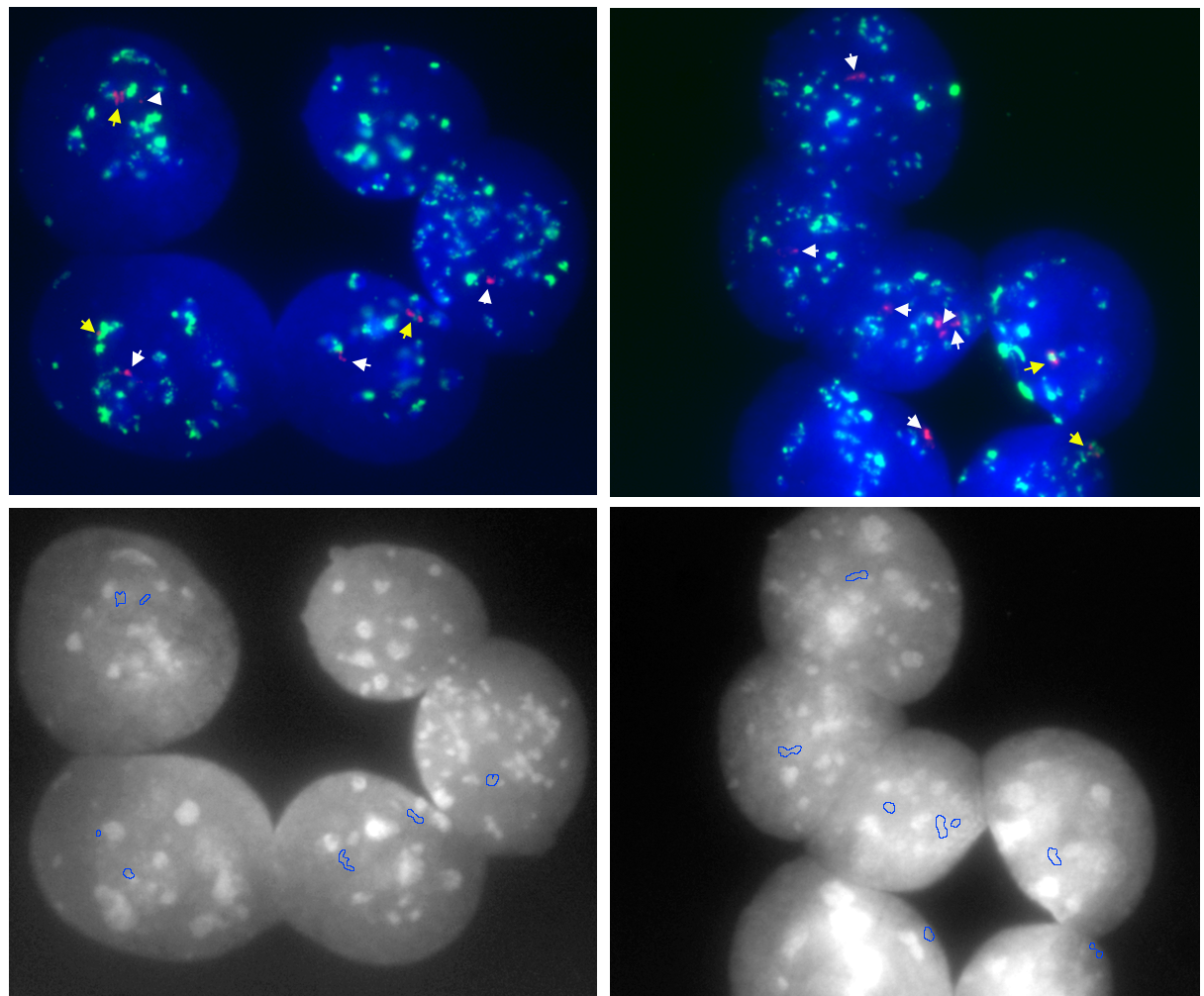

Supplement: Additional file 1 — HAC colocalization with the chromocenters. To shows that SM1-1 (C6) do not colocalize with chromocenters (white arrows), but can colocalize with mouse minor satellite (yellow arrows), several cells from this clone are shown, following hybridization with 17α (red signals) and minor satellite probes (green signals). The bottom panels display the same cells, stained with DAPI to show the chromocenters distribution, and the outline of the HAC in blue. [file 1471-2121-10-18-S1.tiff]
